# Adenoviral oncoprotein E1B55K mediates colocalization of SSBP2 and PML in response to stress

**DOI:** 10.1186/1750-2187-5-6

**Published:** 2010-06-11

**Authors:** Helen B Fleisig, Hong Liang, Lalitha Nagarajan

**Affiliations:** 1 Department of Molecular Genetics, M.D. Anderson Cancer Center, 1515 Holcombe Blvd., Houston TX 77030, USA

## Abstract

Transient expression of adenoviral oncoprotein E1B55K in normal cells induces aggresome formation and sequestration of critical host proteins in aggresomes. Our previous studies reported that Sequence Specific Binding Protein 2 (SSBP2), a candidate tumor suppressor is recruited to aggresomes in adenovirally transformed human embryonal kidney 293 (HEK293) cells. To understand the extent and significance of the E1B55K-SSBP2 interactions in these cells, we have examined SSBP2 localization under conditions of stress in HEK293 cells. SSBP2 localizes to PML- Nuclear Bodies (PML-NBs) in response to inhibition of nuclear export, treatment with etoposide, hydroxyurea or gamma irradiation only in HEK293 cells. Furthermore, the PML-NBs grow in size and number in response to radiation over a 24 hour period in HEK293 cells analogous to previous findings for other cell types. Nonetheless, we conclude that E1B55K subverts SSBP2 function in HEK293 cells. These findings demonstrate the limitations in using HEK293 cells to study DNA damage response and other cellular processes since SSBP2 and similar regulatory proteins are aberrantly localized due to constitutive E1B55K expression.

## Introduction

*Sequence-specific single-stranded DNA binding protein 2 (SSBP2) *was originally isolated as a candidate leukemia suppressor gene from chromosome 5q13.3 that is disrupted in the human acute myelogenous leukemia (AML) cell line ML3 [1]. Inducible expression of *SSBP2 *in AML cells causes growth arrest and partial differentiation [2]. Its effects on differentiation are mediated in part by an evolutionarily conserved direct interaction with the adaptor molecule lim-domain-binding-protein 1(LDB1/CLIM2) in a multi-protein transcriptional complex [3-5].

Localization of the *Drosophila *ortholog ssdp, to the nucleus appeared to be LDB1 dependent under conditions of transient transfection [3-5]. YFP tagged porcine SSBP2, when expressed in Chinese hamster ovary cells localized to mitochondria and translocated to the nucleus when the porcine ortholog of LDB1 was co-expressed[6]. Endogenous human SSBP2 however, localized to punctate structures in the nucleus, nucleolus and cytoplasm in normal cells [7]. Furthermore, nuclear localization of a closely related member SSBP3, is also mediated by phosphorylation of a conserved amino terminal Tyr residue, independent of the LDB1 interaction [7].

HEK293, a cell line routinely used for transient gene expression because of its high transfection efficiency, was originally isolated by transforming human embryonic kidney cells with sheared adenovirus 5 DNA[8]. These cells express adenoviral oncoproteins E1A and E1B55K due to stable integration of the transforming region of the viral genome [8]. In these cells, endogenous SSBP2 is sequestered in aggresomes due to its interaction with E1B55K. This interaction is independent of E1A or other adenovirally encoded proteins as transient expression of E1B55K in human fibroblasts or HeLa cells induced recruitment of endogenous SSBP2 to E1B55K containing aggresomes. Moreover, yeast two hybrid interactions defined E1B55K association to be mediated through the central glycine-proline rich domain of SSBP2[9].

E1B55K, a nuclear cytoplasmic shuttling protein targets tumor suppressor TRP53 and DNA repair pathway proteins of the MRE11 complex for degradation in aggresomes during adenoviral transformation[10-13]. E1B55K mutants lacking nuclear export activity, when expressed in rat kidney cells localize to nuclear aggregates. Furthermore, lack of nuclear export appears to correlate with an augmented oncogenic potential of E1B55K. In addition to TRP53 and E1B55K, the promyelocytic leukemia protein (PML) also localizes within these nuclear aggregates that are similar to PML oncogenic domain (PML-NB or ND10)[14]. Furthermore, inhibition of nuclear export of wild type E1B55K resulted in distribution of PML and TRP53 into PML-NBs [14].

PML recruits a multitude of proteins including DAXX, BLM, SUMO, MRE11 complex into the nuclear structures of PML-NBs with varying composition. Often, multiple proteins are sequestered or released from these structures [15,16]. PML-NBs show a dynamic response to cellular stresses by increased size and number, and altered localization. Based on the SSBP2-E1B55K interactions, we hypothesized that SSBP2 would localize to the PML-NBs in HEK293 cells under conditions of stress. As anticipated, treatment with DNA-damaging agents resulted in the localization of SSBP2 in PML-NBs, demonstrating a tight association between the two proteins.

## Results and Discussion

### SSBP2 colocalizes with PML in HEK 293 cells when nuclear export is blocked

**Figure 1:**
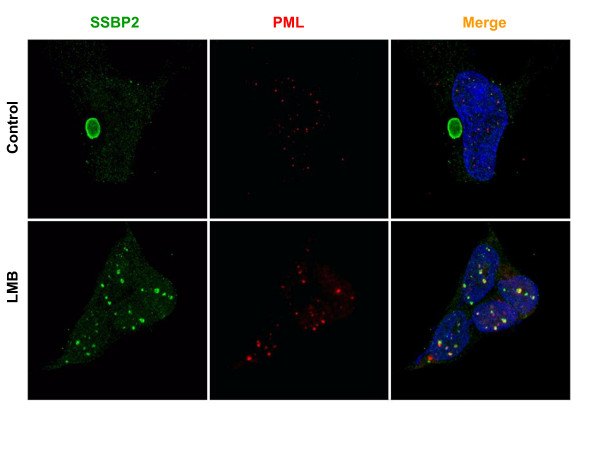
**SSBP2 is localized in the nucleus of HEK293 cells in response to CRM1-export pathway inhibition**. Exponentially growing HEK293 cells were treated with vehicle (upper panel) or 10nM LMB (lower panel) for 3 hrs. prior to fixation and staining with antibodies to SSBP2 [7] or PML (red). Cells were co-stained with DAPI and the images obtained by deconvolution algorithms are shown. In control cells SSBP2 localizes to the large aggresome, while PML is in distinct nuclear bodies (upper panel). When nuclear export is blocked by LMB treatment, SSBP2 and PML localize to nuclear structures (lower panel).

A leucine-rich nuclear export domain (NES) at the N-terminus of E1B55K mediates nucleocytoplasmic shuttling through the CRM1-export pathway [14,17,18]. NES mutants of E1B55K localize to PML-NBs in rat embryonic kidney cells [14]. Previous double-label immunofluorescence experiments from our laboratory demonstrated that inhibition of nuclear export by leptomycin B (LMB) in HEK293 cells resulted in localization of SSBP2 to nuclear inclusions that were distinct from the normal SSBP2 containing punctuate structures [9]. Therefore, we sought to determine whether these nuclear structures are indeed PML-NBs; we examined the localization of both PML and SSBP2 in HEK293 cells treated with CRM1-mediated export inhibitor LMB. Under normal growth conditions SSBP2 was detected predominantly in the E1B55K-containing aggresome with additional weak signals from punctate foci in the nucleus and cytoplasm in HEK293 cells whereas PML localized to distinct PML-NBs (Fig. 1 upper panel). However, when nuclear export of E1B55K was inhibited, the PML nuclear bodies became more prominent and SSBP2 was redistributed into nuclear structures. More importantly, the SSBP2-containing nuclear foci are juxtaposed to PML-NBs with significant overlap (Fig.1 lower panel).

### SSBP2 colocalizes with PML protein in PML-NBs following DNA damage

**Figure 2:**
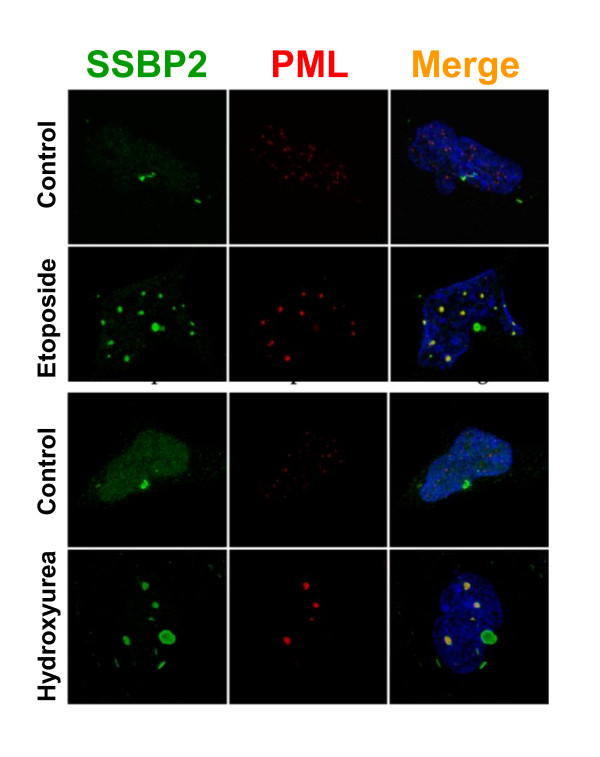
**SSBP2 and PML colocalize in PML-NBs in response to DNA damage in HEK293 cells**. Cells were treated with 1uM etoposide (A) or 0.2 uM hydroxy urea (B) for 24 hours and stained with antibodies to SSBP2 [7] and PML (red). Merged images are shown on the far right. Note the colocalization of SSBP2 and PML only in the PML-NBs in response to DNA damage.

Several DNA repair genes localize to PML-NBs in response to damage signal; PML-NBs are thought to act as DNA damage sensors that undergo dynamic changes including increase in number in response to DNA damage (15).Therefore, we examined whether SSBP2 would colocalize and interact with the PML protein in nuclear foci in response to DNA damage in HEK293 cells. Etoposide induces double stranded breaks by inhibiting topoisomerase II, whereas hydroxyl urea inhibits DNA replication by inhibiting ribonucleotide reductase. As shown in Fig. 2, deconvolution microscopy of cells treated with etoposide or hydroxyl urea for 24 h revealed that SSBP2 colocalized with PML. We also detected residual SSBP2 in aggresomes under both treatment conditions. However, the aggresomes stained only for SSBP2 and not PML as the PML is a nuclear protein that has not been found in E1B55K induced aggresomes.

### Kinetics of PML-NB formation in HEK293 in response to radiation induced DNA damage

**Figure 3:**
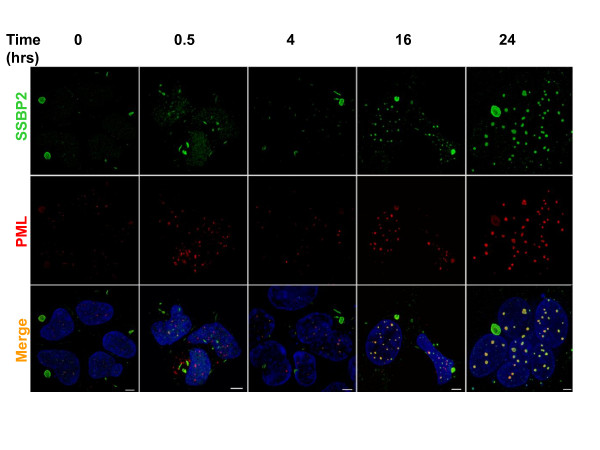
**Kinetics of PML-NB formation in HEK293 cells in response to radiation**. Cultures were exposed to 10gy irradiation and allowed to recover in media at 37°C for 0.5-24 hrs as indicated. Images from SSBP2[7], PML(red) stains and overlay of both (yellow) are depicted. Bars denote 10uM.

**Figure 4:**
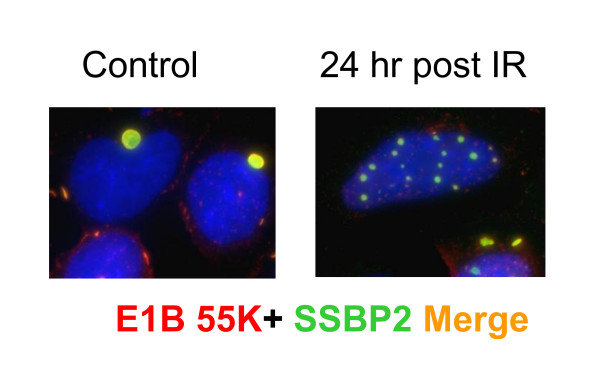
**E1B 55K and SSBP2 are redirected to nuclear bodies after irradiation**. Merged image of E1B55K (red) and SSBP2 [7] shows localization to aggresome in the control HEK293 in contrast to the nuclear bodies 24 hrs after irradiation.

Since radiation induced DNA damage provided a unique system to monitor the kinetics of PML-NB formation, we examined the formation of SSBP2- PML containing nuclear bodies as a function of time in irradiated HEK293 cells (Fig. 3). Thirty minutes after irradiation, the large aggresomes seen at zero time in HEK 293 cells were not readily detectable. More importantly, the beginnings of distinct SSBP2-containing nuclear structures could be recognized. Microbodies containing PML were also readily identified. However, there was no colocalization of the two proteins. By 4 hours, the two proteins were colocalized in some cells. The bodies continued to grow in size to become much larger than the original PML foci, similar to that reported with normal human fibroblasts by Dellaire et al [15]. As early as 16 hours, there was a clear convergence of PML and SSBP2. Once again, there was no detectable PML in the residual aggresome suggesting that these two proteins are juxtaposed only in the nucleus in response to radiation. Fig. 4 shows colocalization of E1B55K and SSBP2 in nuclear bodies of HEK293 cells 24 hours post irradiation. Together, E1B55K SSBP2 and PML appear to be redirected to specific PML-NBs 24 hours after irradiation.

**Figure 5:**
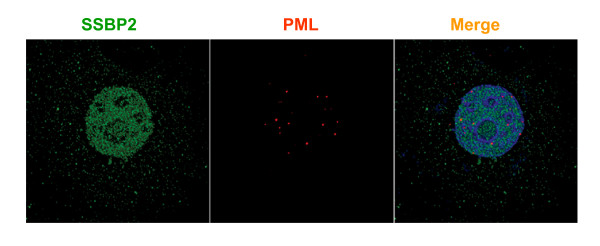
**SSBP2 does not localize to PML-NBs in response to radiation in MCF7 cells**. Cells were exposed to 10gy ionizing radiation and allowed to recover for 7 h. SSBP2 localizes to distinct nuclear structures and cytoplasmic structures. No colocalization with PML is detected.

To determine whether recruitment of SSBP2 into PML-NBs occurs in response to radiation, in cells lacking E1B55K, we examined the breast cancer cells MCF7. These cells express wild type *TRP53 *and have a normal checkpoint response to DNA damaging agent. SSBP2 was not recruited to PML-NBs in response to radiation at multiple time points in MCF7 cells. A representative image at 7 hours after irradiation is shown in fig 5. Similar localization of PML and SSBP2 was also observed in diploid fibroblast cells IMR90 and osteosarcoma cells U2OS after irradiation (data not shown). Thus, the localization of SSBP2 to PML-NBs under conditions of stress is unique to E1B55K expressing HEK293 cells.

PML-containing nuclear bodies are implicated in stress response and DNA repair, although their precise regulatory role is unclear [19-21]. A number of models, that account for multiple functions have been proposed: (i) storage sites for excessive nucleoplasmic proteins, (ii) sites of post translational modification for regulatory proteins (iii) nuclear landmarks for viral replication and early gene transcription and (iv) organizing sites for transcriptional activities [15]. Live cell imaging of GFP tagged PML suggests that stress induced fission results in microstructures which may be involved in reorganizing local chromatin domains[22]. Nonetheless, the mechanisms behind PML-NB generation or potential heterogeneity within the bodies are unknown. The findings reported here reveal for the first time, a partial localization of SSBP2 to PML containing nuclear bodies to be unique to adenovirally transformed HEK293 cells (Figs 1,2,3 and 4). The studies also provide clues as to how smaller PML bodies and E1B55K complexes that are initially juxtaposed assemble subsequently to form larger aggregates (Fig. 3).

*SSBP2 *and its paralogs *SSBP3 *and *4 *were so designated because *CSDP*, the founding member of this family, was isolated as a gene encoding an activity that bound single stranded, pyrimidine rich mirror repeat elements[23]. A potential role for the proline rich central domain of these proteins is to assemble large transcriptional or RNA binding complexes [24]. It would be interesting to determine which of these functions in addition to the LDB1 interaction are disrupted during adenoviral transformation as E1B55K appears to bind the central proline rich domain of SSBP2 [7].

The human *SSBP2 *was originally isolated as a candidate AML suppressor from chromosome 5q, a target of recurrent deletions. Important hallmarks of this leukemia are complex chromosomal translocations and instability. The present results suggest that this novel pathway may be exploited by the adenoviral oncoprotein E1B55K during viral infection and transformation.

HEK293 cells are widely used to study PML function, DNA damage response, due to their robust growth properties and high transfection efficiency. The data presented here underscore the caveats in studying PML-NBs, damage response pathways in this cell line. Moreover, the full repertoire of E1B55K interacting host proteins is presently unknown. Hence, extreme caution is necessary in using these cells as aberrant E1B55K mediated interactions and co-localizations are likely to be misinterpreted as direct interactions.

## Methods

### Cell culture

Two human cell lines were used in the present studies. HEK293, originally isolated in 1977 is an embryonic kidney cell line transformed by adenovirus E1A and E1B genes [9]. This cell line was purchased from Microbix Biosystems Inc., Ontario, Canada. MCF7 cells (breast cancer) were purchased from ATCC (Manassas, Va). Both cell lines were grown in minimum essential medium supplemented with 10% fetal bovine serum at 37°C in 5%CO_2, _95% humidified air.

For the radiation experiments, HEK 293 or MCF7 cells were plated at a density of 2 × 10^5^/well in 6-well plates. This was followed by 10gy of irradiation from a ^137^Cs source (3.7Gy/min) and incubation for 0, 30 minutes, 4, 16 and 24 hours under normal growth conditions.

### Immunoflourescence and deconvolution microscopy

Mouse monoclonal antibody to PML (PG- M3) was purchased from Santa Cruz Biotechnology (Santa Cruz, CA). The rabbit polyclonal antibody against SSBP2 has been characterized previously [2]. The anti-adenovirus 5 E1B55K raised in rat was purchased from Calbiochem. Cells were plated subconfluently on glass coverslips and cultured overnight under exponential growth conditions. To inhibit nuclear export, cells were incubated for 3 h in medium containing 10nM leptomycin B (LMB). Immunofluorescence and deconvolution microscopy was performed as described previously (7). The cells were fixed in 4% paraformaldehyde in phosphate-buffered saline (PBS) for 20 min., followed by permeabilization with 0.2% triton X-100 in PBS. The cells were then washed and incubated in blocking buffer (PBS, 5% NGS, 2% BSA, 0.1%Tween20) for 1 h at room temperature. Primary antibodies were added to the blocking solution for 1 h at room temperature followed by either donkey anti-mouse or donkey anti-rabbit antibodies conjugated to Texas Red or FITC fluorophores (Molecular Probes, In Vitrogen, Carlsbad, CA) for 1 h at room temperature. Cells were post-fixed with 4% PFA, followed by staining with 0.5 μg/ml DAPI. Finally, coverslips were mounted on slides with Prolong antifade mounting medium (Molecular Probes, In Vitrogen, Carlsbad, CA). Metamorph software (Molecular Devices, Dowingtown, Pa.) was used to obtain z-series sections (0.2um) of cells with a 60X/1.45 objective on an inverted microscope (Nikon E2000 U) equipped with a motorized stage, filter wheels and a Photometrics CoolSnap HQ camera (Roper Scientific (Tucson, AZ). Autoquant's AutoDeBlur software (AutoQuant Imaging, Watervliet, NY) was used for deconvolution and visualization of the image stacks.

## Competing interests

The authors declare that they have no competing interests.

## Authors' contributions

HBF conducted the CRM1 pathway inhibition, etoposide and hydroxy urea studies and edited the manuscript. HL conducted the PML-NB formation kinetics experiments. LN took part in the experimental design and wrote the manuscript. All authors read and approved the final manuscript.

## References

[B1] Castro P Liang H Liang JC Nagarajan L A novel, evolutionarily conserved gene family with putative sequence-specific single-stranded DNA-binding activity Genomics 2002 80 1 78 85 10.1006/geno.2002.6805 12079286

[B2] Liang H Samanta S Nagarajan L SSBP2, a candidate tumor suppressor gene, induces growth arrest and differentiation of myeloid leukemia cells Oncogene 2005 24 16 2625 2634 10.1038/sj.onc.1208167 15782145

[B3] Chen L Segal D Hukriede NA Podtelejnikov AV Bayarsaihan D Kennison JA Ogryzko VV Dawid IB Westphal H Ssdp proteins interact with the LIM-domain-binding protein Ldb1 to regulate development Proc Natl Acad Sci USA 2002 99 22 14320 14325 10.1073/pnas.212532399 12381786 PMC137882

[B4] van Meyel DJ Thomas JB Agulnick AD Ssdp proteins bind to LIM-interacting co-factors and regulate the activity of LIM-homeodomain protein complexes in vivo Development 2003 130 9 1915 1925 10.1242/dev.00389 12642495

[B5] Xu Z Meng X Cai Y Liang H Nagarajan L Brandt SJ Single-stranded DNA-binding proteins regulate the abundance of LIM domain and LIM domain-binding proteins Genes Dev 2007 21 8 942 955 10.1101/gad.1528507 17437998 PMC1847712

[B6] Kato Y Kato T Ono T Susa T Kitahara K Matsumoto K Intracellular localization of porcine single-strand binding protein 2 J Cell Biochem 2009 106 5 912 919 10.1002/jcb.22066 19199338

[B7] Fleisig HB Orazio NI Liang H Tyler AF Adams HP Weitzman MD Nagarajan L Adenoviral E1B55K oncoprotein sequesters candidate leukemia suppressor sequence-specific single-stranded DNA-binding protein 2 into aggresomes Oncogene 2007 26 33 4797 4805 10.1038/sj.onc.1210281 17311003

[B8] Dey-Guha I Malik N Lesourne R Love PE Westphal H Tyrosine phosphorylation controls nuclear localization and transcriptional activity of Ssdp1 in mammalian cells J Cell Biochem 2008 103 6 1856 1865 10.1002/jcb.21576 18080319

[B9] Graham FL Smiley J Russell WC Nairn R Characteristics of a human cell line transformed by DNA from human adenovirus type 5 J Gen Virol 1977 36 1 59 74 10.1099/0022-1317-36-1-59 886304

[B10] Araujo FD Stracker TH Carson CT Lee DV Weitzman MD Adenovirus type 5 E4orf3 protein targets the Mre11 complex to cytoplasmic aggresomes J Virol 2005 79 17 11382 11391 10.1128/JVI.79.17.11382-11391.2005 16103189 PMC1193610

[B11] Liu Y Shevchenko A Shevchenko A Berk AJ Adenovirus exploits the cellular aggresome response to accelerate inactivation of the MRN complex J Virol 2005 79 22 14004 14016 10.1128/JVI.79.22.14004-14016.2005 16254336 PMC1280221

[B12] Blackford AN Grand RJ Adenovirus E1B 55-kilodalton protein: multiple roles in viral infection and cell transformation J Virol 2009 83 9 4000 4012 10.1128/JVI.02417-08 19211739 PMC2668481

[B13] Lilley CE Schwartz RA Weitzman MD Using or abusing: viruses and the cellular DNA damage response Trends Microbiol 2007 15 3 119 126 10.1016/j.tim.2007.01.003 17275307

[B14] Endter C Hartl B Spruss T Hauber J Dobner T Blockage of CRM1-dependent nuclear export of the adenovirus type 5 early region 1B 55-kDa protein augments oncogenic transformation of primary rat cells Oncogene 2005 24 1 55 64 10.1038/sj.onc.1208170 15480414

[B15] Dellaire G Ching RW Ahmed K Jalali F Tse KC Bristow RG Bazett-Jones DP Promyelocytic leukemia nuclear bodies behave as DNA damage sensors whose response to DNA double-strand breaks is regulated by NBS1 and the kinases ATM, Chk2, and ATR J Cell Biol 2006 175 1 55 66 10.1083/jcb.200604009 17030982 PMC2064496

[B16] Grisendi S Bernardi R Rossi M Cheng K Khandker L Manova K Pandolfi PP Role of nucleophosmin in embryonic development and tumorigenesis Nature 2005 437 7055 147 153 10.1038/nature03915 16007073

[B17] Kratzer F Rosorius O Heger P Hirschmann N Dobner T Hauber J Stauber RH The adenovirus type 5 E1B-55K oncoprotein is a highly active shuttle protein and shuttling is independent of E4orf6, p53 and Mdm2 Oncogene 2000 19 7 850 857 10.1038/sj.onc.1203395 10702793

[B18] Dosch T Horn F Schneider G Kratzer F Dobner T Hauber J Stauber RH The adenovirus type 5 E1B-55K oncoprotein actively shuttles in virus-infected cells, whereas transport of E4orf6 is mediated by a CRM1-independent mechanism J Virol 2001 75 12 5677 5683 10.1128/JVI.75.12.5677-5683.2001 11356976 PMC114281

[B19] Mirzoeva OK Petrini JH DNA damage-dependent nuclear dynamics of the Mre11 complex Mol Cell Biol 2001 21 1 281 288 10.1128/MCB.21.1.281-288.2001 11113202 PMC88801

[B20] Yang S Kuo C Bisi JE Kim MK PML-dependent apoptosis after DNA damage is regulated by the checkpoint kinase hCds1/Chk2 Nat Cell Biol 2002 4 11 865 870 10.1038/ncb869 12402044

[B21] Barr SM Leung CG Chang EE Cimprich KA ATR kinase activity regulates the intranuclear translocation of ATR and RPA following ionizing radiation Curr Biol 2003 13 12 1047 1051 10.1016/S0960-9822(03)00376-2 12814551

[B22] Ching RW Dellaire G Eskiw CH Bazett-Jones DP PML bodies: a meeting place for genomic loci? J Cell Sci 2005 118 Pt 5 847 854 10.1242/jcs.01700 15731002

[B23] Bayarsaihan D Lukens LN Single-strand-DNA-binding factors specifically recognize the pyrimidine element in the chick alpha2(I) collagen gene promoter Biochem J 1996 314 Pt 1 293 296 8660297 10.1042/bj3140293 PMC1217039

[B24] Neduva V Russell RB Proline-rich regions in transcriptional complexes: heading in many directions Sci STKE 2007 2007 369 1 10.1126/stke.3692007pe1 17228057

